# Supplementary material for: Mapping localization of 21 endogenous proteins in the Golgi apparatus of rodent neurons
Source: Sci Rep. 2023 Feb 18;13:2871. doi: 10.1038/s41598-023-29998-8 (PMC9938882; doi:10.1038/s41598-023-29998-8)
Supplement: Supplementary file 1 — Supplementary Information. [file 41598_2023_29998_MOESM1_ESM.pdf]

Mapping localization of 21 endogenous proteins in the Golgi apparatus of rodent neurons

Danique M van Bommel[1], Ruud F Toonen[1] and Matthijs Verhage*[1,2]

Supplementary information

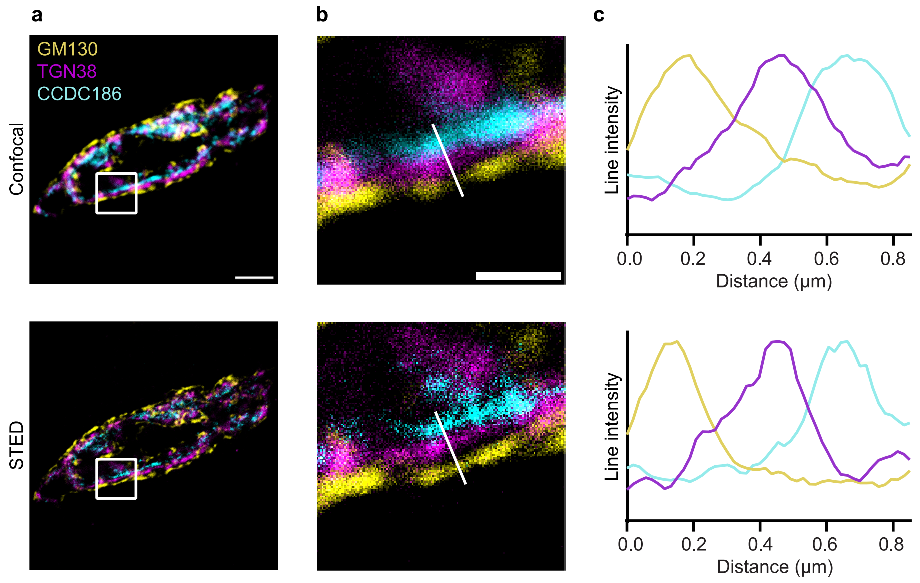

Supplementary Fig. 1 Comparison of confocal and STED microscopy. **a** Confocal and STED image of a neuron stained for GM130 (*cis*-Golgi), TGN38 (TGN) and CCDC186. **b** Zoom from the white square area marked in **a**. **c** Line intensity profile along white line in **b**. Intensities were normalized and smoothed before plotting. Scale bar is 3 μm (**a**) and 1 μm (**b**).

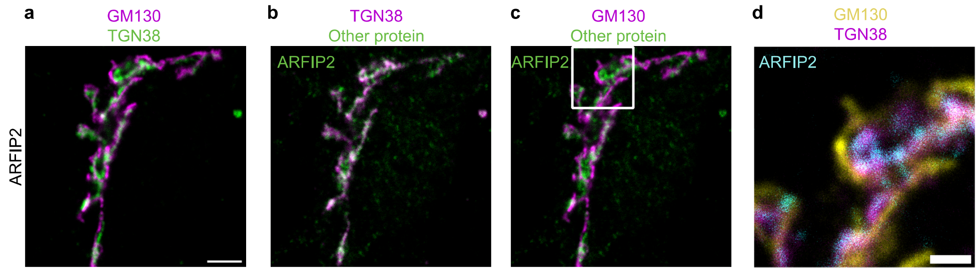

Supplementary Fig. 2 Alternative localization of ARFIP2. **a-c** Representative examples of neurons immunostained for *cis*-Golgi marker GM130, TGN38 and ARFIP2. Images showing immunostaining of *cis*-Golgi and TGN (**a**), TGN and ARFIP2 (**b**), *cis*-Golgi and ARFIP2 (**c**). **d** Zoom from the white square area marked in **c**, showing all three stained proteins. Scale bar is 3 μm (**a**) and 1 μm (**d**).

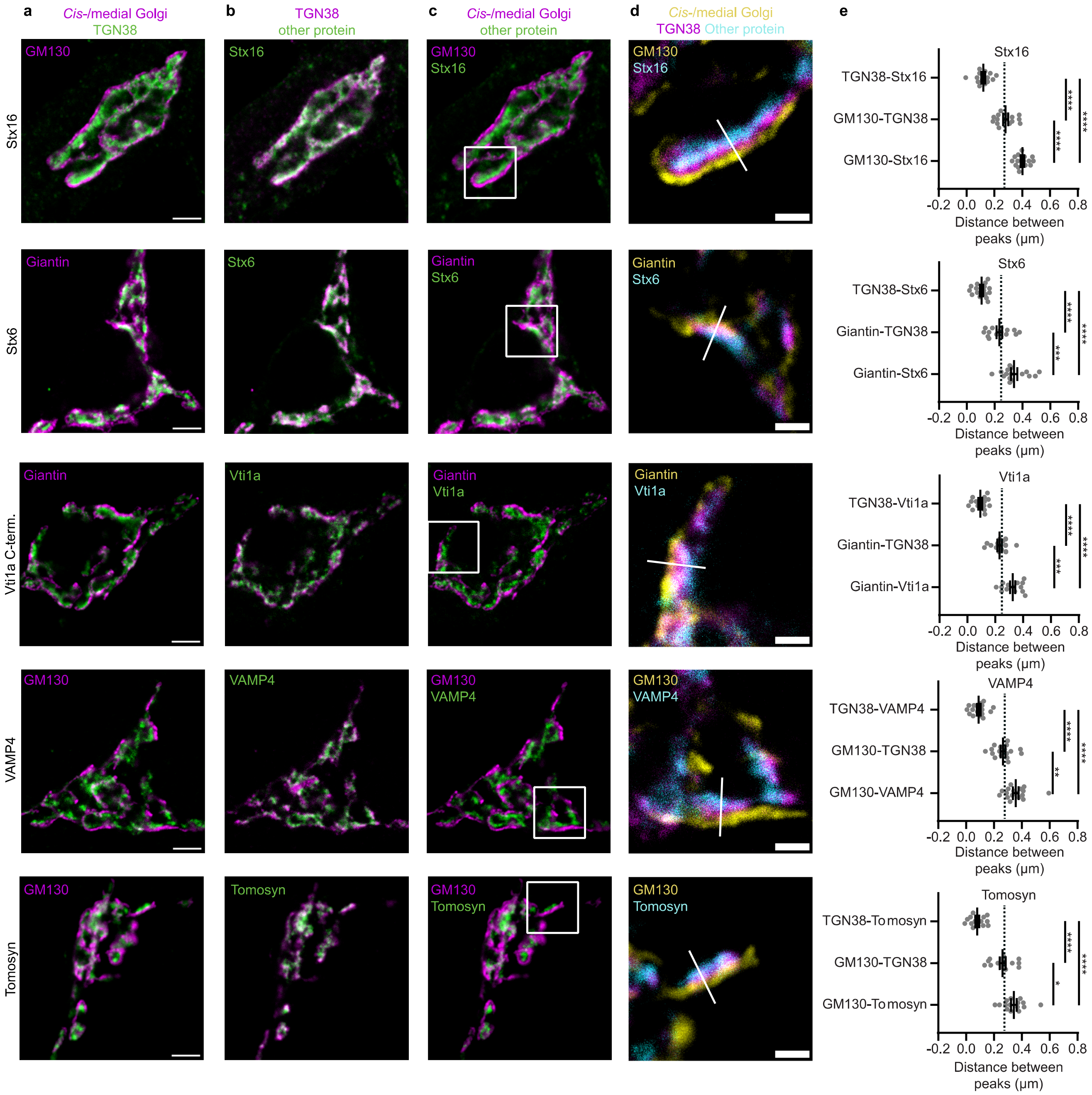

Supplementary Fig. 3 Several Golgi proteins localize downstream of TGN38. **a-c** Representative examples of neurons immunostained for a *cis*-/medial Golgi marker (GM130 or giantin), TGN38 and a protein of interest (Stx16, Stx6, Vti1a C-terminus, VAMP4 and Tomosyn). Images showing immunostaining of *cis*-/medial Golgi marker and TGN (**a**), TGN and protein of interest (**b**), *cis*-/medial Golgi and protein of interest (**c**). **d** Zoom from the white square area marked in **c**, showing all three stained proteins. White line is a representative example of a line drawn for intensity plotting. **e** Distance between intensity peaks of TGN38 and protein of interest, *cis*-/medial Golgi and TGN; and *cis*-/medial Golgi and protein of interest, measured by line scans. Stx16: (TGN38-Stx16: n = 15; GM130-TGN38: n = 15; GM130-Stx16: n = 15). One-way ANOVA with Tukey's multiple comparisons test: TGN38-Stx16 vs GM130-TGN38, TGN38-Stx16 vs GM130-Stx16 and GM130-TGN38 vs GM130-Stx16: ****p < 0.0001. Stx6: (TGN38-Stx6: n = 16; Giantin-TGN38: n = 16; Giantin-Stx6: n = 16). One-way ANOVA with Tukey's multiple comparisons test: TGN38-Stx6 vs Giantin-TGN38 and TGN38-Stx6 vs Giantin-Stx6: ****p < 0.0001, Giantin-TGN38 vs Giantin-Stx6: ***p = 0.0008. Vti1a C-term.: (TGN38-Vti1a C-term.: n = 14; Giantin-TGN38: n = 14; Giantin-Vti1a C-term.: n = 14). One-way ANOVA with Tukey's multiple comparisons test: TGN38-Vti1a C-term. vs Giantin-TGN38 and TGN38-Vti1a C-term. vs Giantin-Vti1a C-term.: ****p < 0.0001, Giantin-TGN38 vs Giantin-Vti1a C-term.: ***p = 0.0005. VAMP4: (TGN38-VAMP4: n = 17; GM130-TGN38: n = 17; GM130-VAMP4: n = 17). One-way ANOVA with Tukey's multiple comparisons test: TGN38-VAMP4 vs GM130-TGN38 and TGN38-VAMP4 vs GM130-VAMP4: ****p < 0.0001, and GM130-TGN38 vs GM130-VAMP4: **p = 0.0011. Tomosyn: (TGN38-Tomosyn: n = 15; GM130-TGN38: n = 15; GM130-Tomosyn: n = 15). One-way ANOVA with Tukey's multiple comparisons test: TGN38-Tomosyn vs GM130-TGN38 and TGN38-Tomosyn vs GM130-Tomosyn: ****p < 0.0001, GM130-TGN38 vs GM130-Tomosyn: *p = 0.0109. Dotted lines represent average distance between TGN38 and GM130 or TGN38 and giantin in the entire dataset. Bars show mean ± SEM. Detailed statistics are shown in Supplementary Table 1. Scale bar is 3 µm (**a**) and 1 µm (**d**).

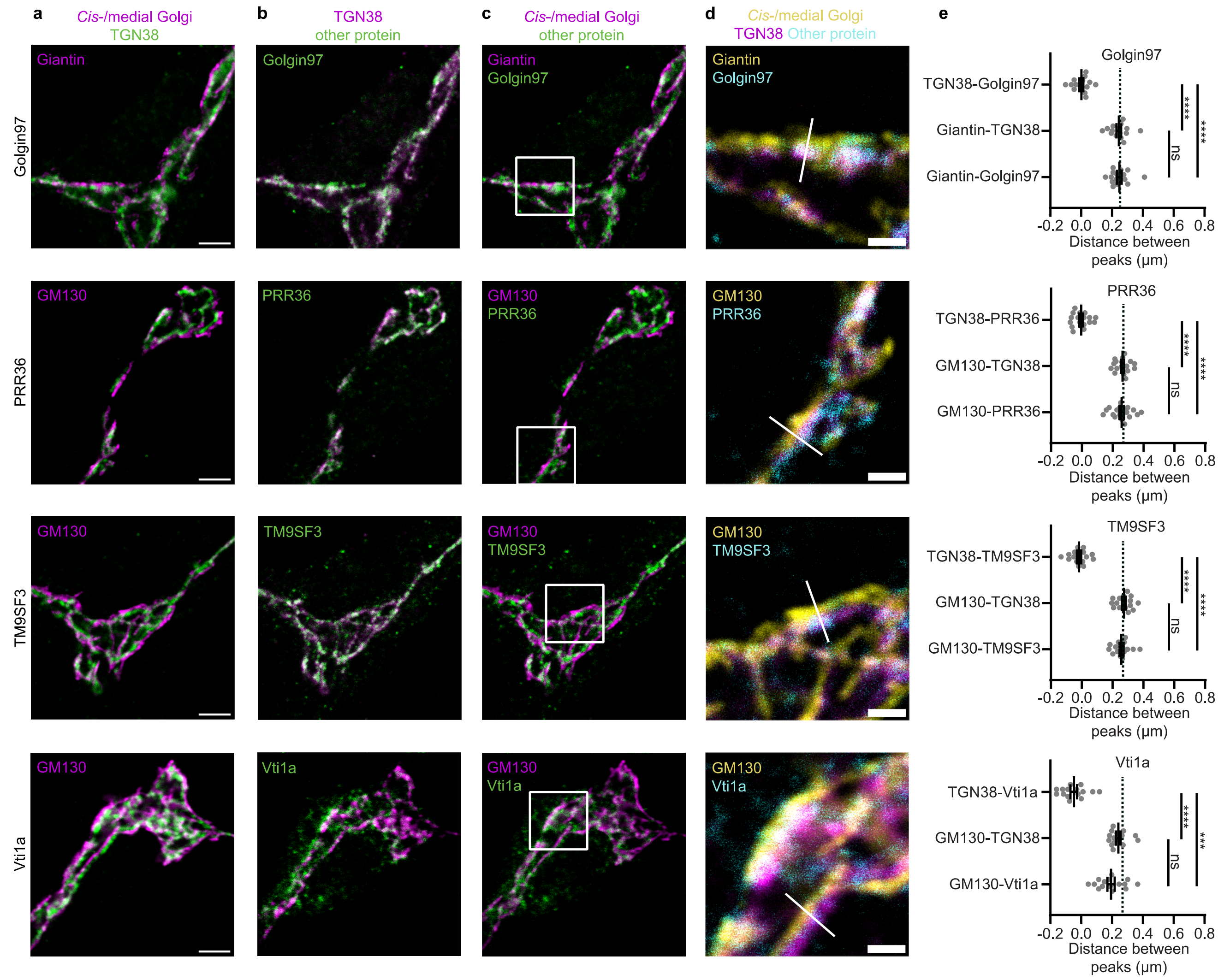

Supplementary Fig. 4 Several Golgi proteins overlap with TGN38. **a-c** Representative examples of neurons immunostained for a *cis*-/medial Golgi marker (GM130 or giantin), TGN38 and a protein of interest (TMEM87A, Golgin97, PRR36 and TM9SF3). Images showing immunostaining of *cis*-/medial Golgi marker and TGN (**a**), TGN and protein of interest (**b**), *cis*-/medial Golgi and protein of interest (**c**). **d** Zoom from the white square area marked in **c**, showing all three stained proteins. White line is a representative example of a line drawn for intensity plotting. **e** Distance between intensity peaks of TGN38 and protein of interest, *cis*-/medial Golgi and TGN; and *cis*-/medial Golgi and protein of interest, measured by line scans. Golgin97: (TGN38-Golgin97: n = 15; Giantin-TGN38: n = 15; Giantin-Golgin97: n = 15). One-way ANOVA with Tukey's multiple comparisons test: TGN38-Golgin97 vs Giantin-TGN38 and TGN38-Golgin97 vs Giantin-Golgin97: ****$p < 0.0001$, Giantin-TGN38 vs Giantin-Golgin97: non-significant (ns). PRR36: (TGN38-PRR36: n = 18; GM130-TGN38: n = 18; GM130-PRR36: n = 18). One-way ANOVA with Tukey's multiple comparisons test: TGN38-PRR36 vs GM130-TGN38 and TGN38-PRR36 vs GM130-PRR36: ****$p < 0.0001$, GM130-TGN38 vs GM130-PRR36: non-significant (ns). TM9SF3: (TGN38-TM9SF3: n = 16; GM130-TGN38: n = 16; GM130-TM9SF3: n = 16). One-way ANOVA with Tukey's multiple comparisons test: TGN38-TM9SF3 vs GM130-TGN38 and TGN38-TM9SF3 vs GM130-TM9SF3: ****$p < 0.0001$, GM130-TGN38 vs GM130-TM9SF3: non-significant (ns). Vti1a: (TGN38-Vti1a: n = 15; GM130-TGN38: n = 15; GM130-Vti1a: n = 15). Kruskall-Wallis test with Dunn's multiple comparisons test: TGN38-Vti1a vs GM130-TGN38: ****$p < 0.0001$, TGN38-Vti1a vs GM130-Vti1a: ***$p = 0.0002$, GM130-TGN38 vs GM130-Vti1a: non-significant (ns). Dotted lines represent average distance between TGN38 and GM130 or TGN38 and giantin in the entire dataset. Bars show mean ± SEM. Detailed statistics are shown in Supplementary Table 1. Scale bar is 3 µm (**a**) and 1 µm (**d**).

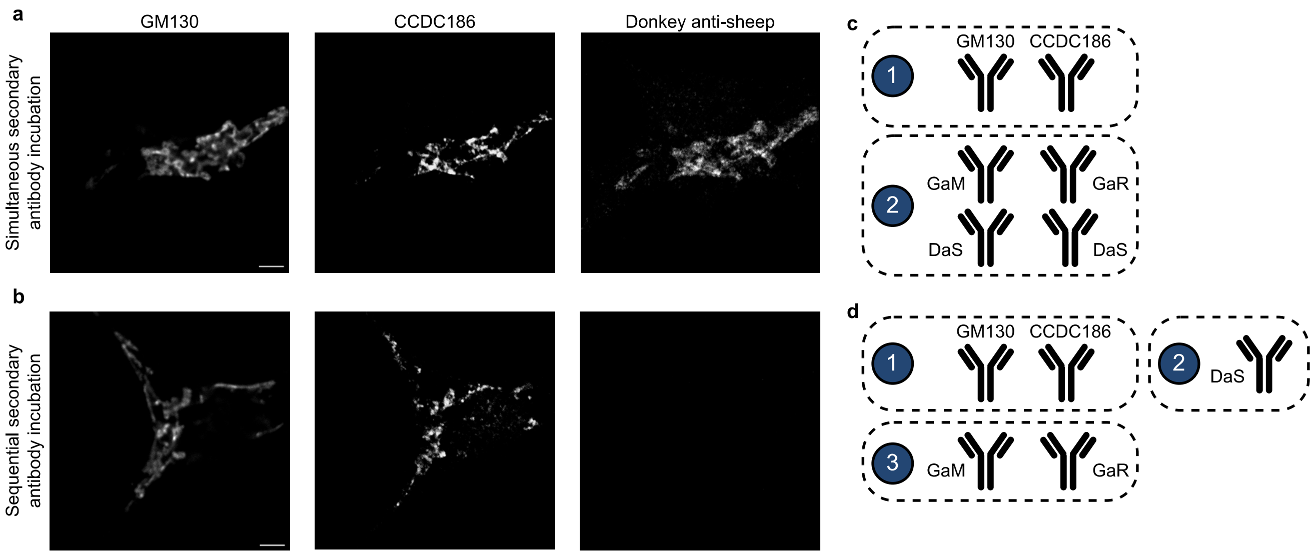

Supplementary Fig. 5 Splitting secondary antibody incubation into two steps prevents cross-reactivity between donkey anti-sheep and goat antibodies. **a-b** Representative examples of neurons immunostained for GM130, CCDC186 and donkey anti-sheep (without a primary sheep antibody). Simultaneous incubation of secondary antibodies causes the anti-sheep antibody to cross-react with the goat secondary antibodies (**a**), while there is no donkey anti-sheep signal after sequential staining (**b**). **c-d** Schematic representation of the protocols. In both protocols, the first step is to incubate primary antibodies against GM130 and CCDC186. In the simultaneous protocol, secondary goat anti-mouse (GaM), goat anti-rabbit (GaR) and donkey anti-sheep (DaS) antibodies are incubated together. This results in the binding of DaS to GaM and GaR antibodies (which bind the antibodies against GM130 and CCDC186, respectively) (**c**). In the sequential protocol, step 2 is incubation of DaS, which does not bind to anything, and step 3 is incubation of GaM and GaR antibodies, which bind the antibodies against GM130 and CCDC186 (**d**). Scale bar is 3 μm.

Supplementary Table 1: Data and statistics. Overview of all data and statistics for each figure. Dataset, condition, average and SEM, the number of independent cells (n), the p-values and statistical tests used are indicated. Statistical tests were two-tailed and used α = 0.05. *p < 0.05; **p < 0.01; ***p < 0.001; ****p < 0.0001. For one-way ANOVA the p-values are also indicated.

Dataset	Condition	Value (Mean ± SEM)	n	p-value	Statistical test
AP1 Figure 2e	(1) TGN38-AP1	0.236 ± 0.0262	14	ns, p = 0.6031: 1 versus 2, ****p <0.0001: 1 versus 3, **p = 0.0036: 2 versus 3	Kruskal-Wallis test (****p < 0.0001) with Dunn's multiple comparisons test
	(2) Giantin-TGN38	0.295 ± 0.0176	14		
	(3) Giantin-AP1	0.531 ± 0.0338	14		
AP3 Figure 2e	(1) TGN38-AP3	0.171 ± 0.0243	16	ns, p = 0.0940: 1 versus 2, ****p <0.0001: 1 versus 3, **p = 0.0042: 2 versus 3	Kruskal-Wallis test (****p < 0.0001) with Dunn's multiple comparisons test
	(2) Giantin-TGN38	0.262 ± 0.0149	16		
	(3) Giantin-AP3	0.433 ± 0.0293	16		
ARFIP2 Figure 2e	(1) TGN38-ARFIP2	0.132 ± 0.0289	18	**p = 0.0018: 1 versus 2, ****p <0.0001: 1 versus 3, ***p = 0.0009: 2 versus 3	One-way ANOVA (****p < 0.0001) with Tukey's multiple comparisons test
	(2) GM130-TGN38	0.256 ± 0.0125	18		
	(3) GM130-ARFIP2	0.388 ± 0.0272	18		
CCDC186 Figure 2e	(1) TGN38-CCDC186	0.123 ± 0.0178	15	****p < 0.0001: 1 versus 2, 1 versus 3, ***p = 0.0004: 2 versus 3	One-way ANOVA (****p < 0.0001) with Tukey's multiple comparisons test
	(2) GM130-TGN38	0.29 ± 0.0163	15		
	(3) GM130-CCDC186	0.412 ± 0.0262	15		
ARL1 Figure 3e	(1) TGN38-ARL1	0.00589 ± 0.0107	15	****p < 0.0001: 1 versus 2, 1 versus 3, ns, p = 0.9277: 2 versus 3	One-way ANOVA (****p < 0.0001) with Tukey's multiple comparisons test
	(2) Giantin-TGN38	0.252 ± 0.0122	15		
	(3) Giantin-ARL1	0.258 ± 0.109	15		
SMIM7 Figure 3e	(1) TGN38-SMIM7	0.00526 ± 0.00888	15	****p < 0.0001: 1 versus 2, 1 versus 3, ns, p = 0.9422: 2 versus 3	One-way ANOVA (****p < 0.0001) with Tukey's multiple comparisons test
	(2) GM130-TGN38	0.271 ± 0.014	15		
	(3) GM130-SMIM7	0.277 ± 0.104	15		
HID1 Figure 3e	(1) TGN38-HID1	0.00454 ± 0.011	16	****p < 0.0001: 1 versus 2, 1 versus 3, ns, p = 0.9744: 2 versus 3	One-way ANOVA (****p < 0.0001) with Tukey's multiple comparisons test
	(2) Giantin-TGN38	0.264 ± 0.0164	16		
	(3) Giantin-HID1	0.269 ± 0.0163	16		
TMEM87A Figure 3e	(1) TGN38-TMEM87A	0.0021 ± 0.0142	15	****p < 0.0001: 1 versus 2, 1 versus 3, ns, p = 0.9942: 2 versus 3	One-way ANOVA (****p < 0.0001) with Tukey's multiple comparisons test
	(2) GM130-TGN38	0.267 ± 0.0157	15		
	(3) GM130-TMEM87A	0.27 ± 0.0134	15		
Giantin Figure 4e	(1) TGN38-Giantin	-0.172 ± 0.0171	16	****p < 0.0001: 1 versus 2, 1 versus 3, 2 versus 3	One-way ANOVA (****p < 0.0001) with Tukey's multiple comparisons test
	(2) GM130-TGN38	0.285 ± 0.0131	16		
	(3) GM130-Giantin	0.113 ± 0.0123	16		
Vti1b Figure 4e	(1) TGN38-Vti1b	-0.138 ± 0.0155	15	****p < 0.0001: 1 versus 2, **p = 0.0025: 1 versus 3, *p = 0.0201: 2 versus 3	Kruskal-Wallis test (****p < 0.0001) with Dunn's multiple comparisons test
	(2) GM130-TGN38	0.292 ± 0.0185	15		
	(3) GM130-Vti1b	0.154 ± 0.0188	15		
CASC4 Figure 4e	(1) TGN38-CASC4	-0.0854 ± 0.00972	15	****p < 0.0001: 1 versus 2, 1 versus 3, ns, p = 0.9610: 2 versus 3	One-way ANOVA (****p < 0.0001) with Tukey's multiple comparisons test
	(2) GM130-TGN38	0.278 ± 0.0127	15		
	(3) GM130-CASC4	0.192 ± 0.016	15		
Distance from TGN38 Figure 5	GM130	-0.272 ± 0.00442	175		
	Giantin	-0.246 ± 0.00693	108		
	Vti1b	-0.138 ± 0.0155	15		
	CASC4	-0.0854 ± 0.00972	15		
	Vti1a	-0.0473 ± 0.0209	15		

	TM9SF3	-0.0162 ± 0.0138	16		
	PRR36	-0.00491 ± 0.012	18		
	Golgin97	0.00189 ± 0.0131	15		
	TMEM87A	0.0021 ± 0.0142	15		
	SMIM7	0.00526 ± 0.00888	15		
	HID1	0.00454 ± 0.011	16		
	ARL1	0.00589 ± 0.0107	15		
	Tomosyn	0.0808 ± 0.0138	15		
	VAMP4	0.0908 ± 0.0127	17		
	Vti1a C-terminus	0.0947 ± 0.0136	14		
	Stx6	0.106 ± 0.0121	16		
	Stx16	0.121 ± 0.0138	15		
	CCDC186	0.123 ± 0.0178	15		
	ARFIP2	0.132 ± 0.0289	18		
	AP3	0.171 ± 0.0243	16		
	AP1	0.236 ± 0.0262	14		
Stx16 Sup. Figure 2e	(1) TGN38-Stx16	0.121 ± 0.0138	15	****p < 0.0001: 1 versus 2, 1 versus 3, 2 versus 3	One-way ANOVA (****p < 0.0001) with Tukey's multiple comparisons test
	(2) GM130-TGN38	0.281 ± 0.0175	15		
	(3) GM130-Stx16	0.402 ± 0.0123	15		
Stx6 Sup. Figure 2e	(1) TGN38-Stx6	0.106 ± 0.0121	16	****p < 0.0001: 1 versus 2, 1 versus 3, ***p = 0.0008: 2 versus 3	One-way ANOVA (****p < 0.0001) with Tukey's multiple comparisons test
	(2) Giantin-TGN38	0.235 ± 0.02	16		
	(3) Giantin-Stx6	0.34 ± 0.0231	16		
Vti1a C-terminus Sup. Figure 3e	(1) TGN38-Vti1a	0.0947 ± 0.0136	14	****p < 0.0001: 1 versus 2, 1 versus 3, ***p = 0.0005: 2 versus 3	One-way ANOVA (****p < 0.0001) with Tukey's multiple comparisons test
	(2) Giantin-TGN38	0.232 ± 0.0159	14		
	(3) Giantin-Vti1a	0.326 ± 0.0188	14		
VAMP4 Sup. Figure 3e	(1) TGN38-VAMP4	0.0908 ± 0.0127	17	****p < 0.0001: 1 versus 2, 1 versus 3, **p = 0.0011: 2 versus 3	One-way ANOVA (****p < 0.0001) with Tukey's multiple comparisons test
	(2) GM130-TGN38	0.266 ± 0.017	17		
	(3) GM130-VAMP4	0.357 ± 0.0198	17		
Tomosyn Sup. Figure 3e	(1) TGN38-Tomosyn	0.0808 ± 0.0138	15	****p < 0.0001: 1 versus 2, 1 versus 3, *p = 0.0109: 2 versus 3	One-way ANOVA (****p < 0.0001) with Tukey's multiple comparisons test
	(2) GM130-TGN38	0.262 ± 0.0207	15		
	(3) GM130-Tomosyn	0.343 ± 0.0209	15		
Golgin97 Sup. Figure 4e	(1) TGN38-Golgin97	0.00189 ± 0.0131	15	****p < 0.0001: 1 versus 2, 1 versus 3, ns, p = 0.9955: 2 versus 3	One-way ANOVA (****p < 0.0001) with Tukey's multiple comparisons test
	(2) Giantin-TGN38	0.246 ± 0.0154	15		
	(3) Giantin-Golgin97	0.247 ± 0.0159	15		
PRR36 Sup. Figure 4e	(1) TGN38-PRR36	-0.00491 ± 0.012	18	****p < 0.0001: 1 versus 2, 1 versus 3, ns, p = 0.9610: 2 versus 3	One-way ANOVA (****p < 0.0001) with Tukey's multiple comparisons test
	(2) GM130-TGN38	0.263 ± 0.0105	18		
	(3) GM130-PRR36	0.258 ± 0.0157	18		
TM9SF3 Sup. Figure 4e	(1) TGN38-TM9SF3	-0.0162 ± 0.0138	16	****p < 0.0001: 1 versus 2, 1 versus 3, ns, p = 0.6640: 2 versus 3	One-way ANOVA (****p < 0.0001) with Tukey's multiple comparisons test
	(2) GM130-TGN38	0.275 ± 0.0127	16		
	(3) GM130-TM9SF3	0.259 ± 0.0131	16		
Vti1a Sup. Figure 4e	(1) TGN38-Vti1a	-0.0473 ± 0.0209	15	****p < 0.0001: 1 versus 2, ***p = 0.0002: 1 versus 3, ns, p = 0.6479: 2 versus 3	Kruskal-Wallis test (****p < 0.0001) with Dunn's multiple comparisons test
	(2) GM130-TGN38	0.241 ± 0.0148	15		
	(3) GM130-Vti1a	0.194 ± 0.0232	15		
